# Prevalence of pterygium in a population in Northern Japan: the Locomotive Syndrome and Health Outcome in Aizu Cohort Study

**DOI:** 10.1111/aos.12044

**Published:** 2013-05

**Authors:** Takatoshi Tano, Koichi Ono, Yoshimune Hiratsuka, Koji Otani, Miho Sekiguchi, Shinichi Konno, Shinichi Kikuchi, Yoshihiro Onishi, Misa Takegami, Masakazu Yamada, Shunichi Fukuhara, Akira Murakami

**Affiliations:** 1 Department of Ophthalmology, Juntendo University School of MedicineTokyo, Japan; 2 Department of Ophthalmology, Juntendo Tokyo Koto Geriatric Medical CenterTokyo, Japan; 3 National Institute of Public HealthTokyo, Japan; 4 Department of Orthopaedic Surgery, Fukushima Medical University School of MedicineFukushima, Japan; 5 Department of Epidemiology and Healthcare Research, Graduate School of Medicine and Public Health, Kyoto UniversityKyoto, Japan; 6 Institute for Health Outcomes & Process Evaluation ResearchKyoto, Japan; 7 Division for Vision Research, National Institute of Sensory Organs, National Tokyo Medical CenterTokyo, Japan

**Keywords:** Japanese, population-based study, prevalence, pterygium, risk factors

## Abstract

**Purpose:**

The aim of the study was to determine the prevalence and risk factors for pterygium in a population aged 40–74 years in Fukushima Prefecture, Japan.

**Methods:**

Of 4185 citizens of the towns of Minamiaizu-machi and Tadami-machi, 2312 (55.2%) gave consent to an ocular examination during a health examination. Pterygium was diagnosed when a radially oriented fibrovascular lesion growing over the limbus into the cornea was observed. Eyes with a history of pterygium excision were also diagnosed with pterygium. Prevalence and factors associated with pterygium were investigated.

**Results:**

Of the 2312 subjects, 101 (4.4%; 95% confidence interval, 3.6–5.3%) had pterygium in at least 1 eye. The average age (±SD) of the subjects was 64.3 ± 8.0 years. Gender, age, outdoor job history and smoking history were examined as possible associated factors, but only age was found to be significantly associated with pterygium in logistic regression analysis.

**Conclusion:**

The prevalence of pterygium was 4.4% in the study population. This low rate may be due to the northern latitude of these towns. Age was associated with a risk of pterygium, but gender and outdoor job history were not associated with onset of pterygium in this study.

## Introduction

Pterygium is a triangular fibrovascular tissue that develops from the bulbar conjunctiva and grows towards and over the corneal limbus (Jaros & Deluise 1988). The tissue may require surgical excision if it affects vision by encroaching on the visual axis or causing irregular cornea astigmatism (Jaros & Deluise 1988). Many theories have been advanced to explain the cause of pterygium, but the definitive causative mechanism is unknown. The prevalence of pterygium varies according to age and sex (Moran & Hollows 1984), geographical location (Detels & Dhir 1967), ultraviolet (UV) sunlight exposure (Taylor et al. 1989) and occupation (Karai & Horiguchi 1984). Environmental factors play an important role in the development of many diseases, and a higher prevalence of pterygium has been reported at lower latitudes and in countries with high UV exposure (Taylor et al.1989; McCarty et al. 2000; Shiroma et al. 2009). However, the rate of pterygium does not always correlate with latitude (Shiroma et al. 2009), as in the same area, the effect of UV may vary due to individual protection and lifestyle (McCarty et al. 2000). Occupations and activities with high UV exposure, such as welding (Karai & Horiguchi 1984) and outdoor work (Cajucom-Uy et al. 2010), have also been associated with a higher rate of pterygium. There is also a generally higher prevalence in rural regions than in urban regions (Panchapakesan et al. 1998; Wong et al. 2001; Gazzard et al. 2002; Wu et al. 2002; Ma et al. 2007). The pathogenesis may involve heat, microtrauma, a pterygium angiogenesis factor, chronic inflammation, genetic predisposition and local eye stimulation by UV, as reviewed by Coroneo (1993). Previous studies (Shiroma et al. 2009; West & Munoz 2009; Cajucom-Uy et al. 2010) have also shown that onset of pterygium is only weakly associated with body height, body weight, hypertension, hyperlipidaemia and diabetes mellitus.

The Kumejima Study was the first Japanese population-based study of the prevalence of pterygium (Shiroma et al. 2009). This study was performed in the southernmost region of Japan, and to our knowledge, a similar study has not been conducted in the adult population in a northern rural region of Japan. Therefore, the aims of this study were to examine the prevalence of and risk factors for pterygium in a northern rural region of Japan and to compare the results with those found in the Kumejima Study and in other populations.

## Subjects and Methods

### Study population

The Locomotive Syndrome and Health Outcome in Aizu Cohort Study (Otani et al. 2012) is an ongoing prospective study of locomotive disorders, health outcomes and common eye diseases in Japanese subjects aged 40–74 years. The subjects were residents of Minamiaizu-machi or Tadami-machi who completed a specified health examination. This examination is provided by insurance organizations such as the National Health Insurance Society to people aged 40–74 years who have the appropriate medical insurance. The objectives of the examination are to protect against and improve metabolic syndrome, which is strongly associated with lifestyle-related diseases, and to provide health guidance to people found to have metabolic syndrome or premetabolic syndrome. The subjects of this study all underwent this examination.

The overall design, survey methods and procedures of the study have been described elsewhere (Otani et al. 2012). Briefly, locomotory and funduscopic examinations were carried out only for residents who received an explanation of this study, gave their agreement and submitted written informed consent when taking the specified medical examination. The study was conducted between 13 April and 17 June 2009. The prevalence of pterygium was examined as part of a population-based epidemiologic survey on ocular diseases. A total of 2851 residents of Minamiaizu and 1334 residents of Tadami aged 40–74 years were invited to participate. The baseline participants (*n* = 2312) represented 55.2% of the eligible residents in the two areas. The study was conducted according to the recommendations of the Declaration of Helsinki and was approved by the institutional review board at Juntendo University School of Medicine, Japan.

Minamiaizu-machi and Tadami-machi are located in north-eastern Japan, at longitude 139°46′N, latitude 37°12′E; and longitude 139°18′N, latitude 37°21′E, respectively. The towns are adjoining and have areas of approximately 745 and 886 km^2^, respectively. Most residents have remained in the two towns for many years, making this community appropriate for a cohort study. The weather in the region is cool, with average daily temperatures of 9.7°C in Minamiaizu-machi and 10.6°C in Tadami-machi, and the yearly total rainfall is 1020 and 1193 mm, respectively. Both towns have heavy snowfall, especially in Tadami-machi, where about 55% of the yearly total rainfall is snow.

### Examinations

The specified medical examination included six components: (i) a questionnaire on medication history, smoking history, occupation and history of outdoor work; (ii) measurement of height, body weight, abdominal circumference and BMI; (iii) physical examinations; (iv) blood pressure; (v) haematological tests for triglyceride, HDL cholesterol, LDL cholesterol, blood glucose, and fasting blood glucose or HbA1c, and tests for hepatic function; and (vi) urinalysis.

The first and current occupations of the subjects were investigated through classification into 10 groups: (i) clerical work; (ii) managerial work; (iii) professional and technological work, (iv) sales; (v) service; (vi) security service; (vii) agriculture, forestry and fisheries; (viii) transportation and telecommunications; (ix) manufacturing and labouring and 10 others. Subjects with a work history in agriculture, forestry and fisheries were determined to be those with a history of outdoor work.

Locomotory and ocular examinations were then conducted in subjects who submitted written informed consent to this study. A detailed ophthalmic screening examination was performed, including digital colour fundus photographs (45°) taken using a nonmydriatic ocular fundus camera system (Nonmyd α-D III; Kowa Inc., Nagoya, Japan). Pterygium was diagnosed when a radially oriented fibrovascular lesion growing over the limbus into the cornea was observed with penlight (Doctor-Light; Neitz Instruments Co., Japan). Eyes with a history of pterygium excision were also diagnosed with pterygium. Eyes with an atypical shape and invading tissue, symblepharon and conjunctival scar tissue, and a history of ocular trauma were diagnosed with pseudopterygium and were not included as cases of pterygium. More than 99% of the diagnoses were made by the same ophthalmologist.

### Data analysis

All data were stored at Juntendo University and analysed using SPSS16.0 J for Windows (SPSS Japan Inc, Tokyo, Japan). An unpaired *t*-test and chi-square test were conducted to compare age, gender, outdoor job history and smoking history in subjects with and without pterygium. Risk factors analysed for an association with onset of pterygium included gender, age, smoking history and outdoor work history, because these factors have previously been linked to pterygium in many studies (Karai & Horiguchi 1984; Moran & Hollows 1984; Taylor et al.1989; McCarty et al. 2000; Shiroma et al. 2009; Cajucom-Uy et al. 2010). Body height, body weight, hypertension, hyperlipidaemia, and diabetes mellitus were excluded, because these factors have previously been shown to have weak associations with pterygium. The prevalence of pterygium was calculated by direct age standardization of the population of Minamiaizu-machi and Tadami-machi. Subjects were stratified into 10-year age groups, and odds ratios for all age groups were calculated in multivariate logistic regression analyses. For analysis based on clinical grades of pterygium, a case with bilateral pterygium was classified according to the higher graded eye (the worse eye). Significant variables (p < 0.05) in univariate analysis were used in multivariate logistic regression analyses. The odds ratio (OR) and 95% confidence interval (CI) for an association with pterygium were calculated from the logistic regression model.

## Results

**Fig. 1:**
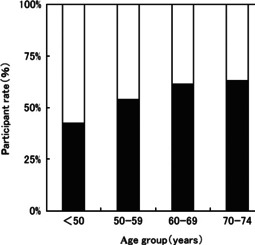
Participation rate by age group. The participation rate was higher in older age groups. □ Nonparticipant, ▪ participant.

Of the 4185 eligible residents, 2312 (55.2%) underwent the eye examination. The participation rate was higher in the older age groups (Fig. 1). The 2312 subjects had an average age of 64.3 ± 8.0 years. Women were more common among the subjects compared with the 1734 nonparticipants (male-to-female ratio 1009:1442 versus 993:741, p < 0.001, chi-square test). Two of the 2312 participants had artificial left eyes, and therefore, 2312 right eyes and 2310 left eyes were included in the analysis.

Distributions of age, gender, outdoor job history and smoking history in subjects with and without pterygium are shown in Table 1. The average age of subjects with pterygium in at least 1 eye was 66.7 ± 7.4 years, and the average age of those with bilateral pterygium was 68.6 ± 6.6 years. These groups had a significantly higher age than subjects without any pterygium and without bilateral pterygium, respectively. Gender, outdoor job history and smoking history showed no significant differences between subjects with and without pterygium in at least 1 eye or between those with and without bilateral pterygium. Five patients were diagnosed with pseudopterygium based on an interview and consultation. The cause was trauma in three patients, and the condition occurred after herpetic keratitis in one case and for an unknown reason (but probably after keratitis) in one case. These patients were excluded from further analysis.

**Table 1:** Characteristics of subjects with and without pterygium

Pterygium	Subjects with any pterygium† (*n* = 101)	Subjects without any pterygium (*n* = 2211)	p-value	Subjects with bilateral pterygium‡ (*n* = 36)	Subjects without bilateral pterygium (*n* = 2276)	p-value
Age (years)	66.7 ± 7.4	64.2 ± 8.0	0.002	68.6 ± 6.6	64.2 ± 8.0	0.001
Gender (male/female)	37/64	906/1305	0.444	14/22	929/1347	0.950
Outdoor job history*	29.7%	22.3%	0.108	36.1%	22.5%	0.082
Smoking history	12.9%	14.3%	0.795	11.1%	14.3%	0.762

* Outdoor jobs include full-time and part-time outdoor jobs during the lifetime of the subject.

† Any pterygium is defined as pterygium in either eye or both eyes.

‡ Bilateral pterygium is defined as pterygium in both eyes.

p-value for subjects with pterygium versus without pterygium (unpaired *t*-test or chi-square test).

Prevalences of pterygium for men and women classified by age, outdoor job history and smoking history are shown in Table 2. There were 101 subjects (4.4%; 95% CI, 3.6–5.3%) with pterygium in at least one eye, including 36 (1.6%; 95% CI, 1.1–2.1%) with bilateral pterygium. The highest prevalence occurred in the 70–74 age group, and the prevalence did not differ between genders. The prevalence increased with age, but with no significant difference among age groups. Gender, age, outdoor job history and smoking history were examined as potential risk factors in univariate analysis, but showed no significant association with pterygium (p > 0.05).

**Table 2:** Prevalence of pterygium by gender

Pterygium	Men (95% CI)	Women (95% CI)	Total (95% CI)
Any pterygium*	3.9% (2.8–5.4)	4.7% (3.6–5.9)	4.4% (3.6–5.3)
Bilateral pterygium†	1.5% (0.8–2.5)	1.6% (1.0–2.4)	1.6% (1.1–2.1)
Age			
<50	2.6% (0.3–9.2)	3.2% (0.7–9.0)	2.9% (1.0–6.7)
50–59	1.7% (0.3–4.8)	4.5% (2.3–8.0)	3.3% (1.8–5.5)
60–69	4.4% (2.7–6.8)	3.4% (2.1–5.1)	3.8% (2.8–5.1)
70–74	5.1% (2.7–8.7)	7.3% (4.9–10.3)	6.5% (4.6–8.7)
Outdoor job history			
+	5.2% (2.5–9.4)	6.0% (3.7–9.2)	5.7% (3.9–8.1)
−	3.6% (2.4–5.2)	4.2% (3.1–5.7)	4.0% (3.1–5.0)
Smoking history			
+	3.3% (1.5–6.1)	7.4% (2.1–17.9)	4.0% (2.1–6.7)
−	4.2% (2.8–6.0)	4.6% (3.5–5.8)	4.4% (3.6–5.4)

CI = confidence interval.

* Any pterygium is defined as pterygium in either eye or both eyes.

† Bilateral pterygium is defined as pterygium in both eyes.

In multiple logistic regression analysis, older age (OR = 1.36 for each 10-year increase) was associated with pterygium. Male gender (OR = 0.83), outdoor job history (OR = 1.22) and smoking history (OR = 1.14) were not associated with pterygium (Table 3). In a similar analysis, older age (OR = 1.90 for each 10-year increase) was associated with bilateral pterygium. Male gender (OR = 0.92), outdoor job history (OR = 1.95) and smoking history (OR = 0.75) were not associated with bilateral pterygium.

**Table 3:** Logistic regression analysis of factors with a possible association with pterygium

	Any pterygium*	Odds ratio (95% CI)	Bilateral pterygium†	Odds ratio (95% CI)
				
	Odds ratio	Adjusted odds ratio	Odds ratio	Adjusted odds ratio
Age (years)				
Age per 10	1.41 (1.09,1.8)	1.36 (1.04,1.78)	1.90 (1.20,3.01)	1.78 (1.10,2.89)
Gender				
Male	0.83 (0.55,1.26)	0.83 (0.53,1.3)	0.92 (0.47,1.81)	0.98 (0.48,2.02)
Female	1 (–)	1 (–)	1 (–)	1 (–)
Outdoor job history‡				
+	1.47 (0.95,2.28)	1.22 (0.77,1.93)	1.95 (0.98,3.88)	1.43 (0.69,2.94)
−	1 (–)	1 (–)	1 (–)	1 (–)
Smoking history				
+	0.88 (0.49,1.60)	1.14 (0.60,2.18)	0.75 (0.26,2.13)	1.00 (0.33,3.09)
−	1 (–)	1 (–)	1 (–)	1 (–)

CI = confidence interval.

* Any pterygium is defined as pterygium in either eye or both eyes.

† Bilateral pterygium is defined as pterygium in both eyes.

‡ Outdoor jobs included full-time and part-time outdoor jobs during the lifetime of the subject.

## Discussion

The study showed that 4.4% of the Japanese population aged 40–74 years in a northern rural region had pterygium in at least one eye and 1.6% had bilateral pterygium. The prevalence of pterygium has ranged from 2.8% to 33.0% in previous population-based studies (McCarty et al. 2000; Wu et al. 2002). A prevalence of 33.0% was found in a Chinese population aged ≥50 years in Doumen County, Southern China (Wu et al. 2002), and a rate of 30.9% was found in a Japanese population aged ≥40 years in a south-western island of Japan (The Kumejima Study; Shiroma et al. 2009). In contrast, a prevalence of 2.83% was found in a Caucasian population aged ≥40 years in Victoria, Australia (McCarty et al. 2000), and a rate of 6.9% was found in a Chinese population aged ≥40 years in Singapore (Wong et al. 2001).

The relatively low prevalence of 4.4% in this study is similar to that in the study in the Australian state of Victoria (McCarty et al. 2000), which is of interest because the latitudes of the regions of the two studies are similar (Minamiaizu-machi and Tadami-machi, 37°N; Victoria, 37°S). Cameron and others have proposed the presence of a ‘pterygium belt’ located at 37° north and south of the equator within which pterygium prevalence increases with greater proximity to the equator (Cameron 1965; Detels & Dhir 1967). The locations in the two studies are at similar latitudes that are farthest from the equator in the pterygium belt, which may account for the low and similar prevalence of pterygium. However, the prevalence may not necessarily be related only to latitude (Panchapakesan et al. 1998; Wong et al. 2001; Gazzard et al. 2002; Wu et al. 2002; Ma et al. 2007) because each study differs in the number of subjects, race, age distribution, lifestyle and occupation.

One limitation of the current study was that the response rate was low (55.2% of eligible participants), which could have caused significant selection and information bias that could lead to under- or overestimation of relationships. The subjects of the health examination were persons aged 40–74 years who enrolled in the National Health Insurance system. Enrolment is not compulsory, but is voluntary. Consequently, basic data for persons who did not enrol in the National Health Insurance are not available. The mean percentage of this population who took the health examination specified by the Ministry of Health, Labour and Welfare in Japan was 43.3% in fiscal year 2010 (http://www.mhlw.go.jp/stf/houdou/2r98520000024j2g-att/2r98520000024j3x.pdf).

Disease prevention is limited because this percentage is similar every year. Various kinds of bias in data collection from the questionnaire (for example, on job history used for identifying outdoor workers) may also have influenced the results. Also, patients were diagnosed with pterygium by an ophthalmologist using pen light alone; therefore, it is possible that extremely small pterygium may have been overlooked.

In the analysis of age groups, we found that age was a significant risk factor for the development of pterygium, a finding that is consistent with most previous studies (Panchapakesan et al. 1998; McCarty et al. 2000; Luthra et al. 2001; Wong et al. 2001; Ma et al. 2007; Lu et al. 2009; Cajucom-Uy et al. 2010), although one study found that age was not a significant factor (West & Munoz 2009). Age was also significant in a multiple logistic regression model (OR = 1.36 for each 10-year increase). Pterygium rarely remits once it develops. Moreover, eyes with a history of pterygium excision were also diagnosed with pterygium in the current study and in previous studies. Older age cohorts may also have higher levels of sun/UV-B exposure because of less use of sunglasses, which may also account for the significant association of age with pterygium (McCarty et al. 2000). Overall, the results of this and previous studies indicate that it is very likely that the prevalence of pterygium increases with age.

The association of gender with development of pterygium is controversial. Various studies have reported that men are at higher risk than women (Panchapakesan et al.1998; McCarty et al. 2000; Wong et al. 2001; Ma et al. 2007; West & Munoz 2009; Cajucom-Uy et al. 2010), whereas other studies have found no gender difference (Luthra et al. 2001) and a higher risk for women (Wu et al. 2002). Previous studies have shown that outdoor work is significantly more common in subjects with pterygium than in those without pterygium (McCarty et al. 2000; Luthra et al. 2001), with cumulative exposure to UV radiation playing a significant role, and one study showed that women have significantly lower cumulative exposure to UV radiation than men (West et al. 1998). It has also been suggested that mean ocular sun exposure is significantly higher in subjects with pterygium and that there is a strong relationship between lifetime ocular sun exposure and lifetime ocular UV-B exposure (West et al. 1998).

The relationship between the risk of development of pterygium and gender may also be complicated by lifestyle. In the present study, men had a slightly smaller odds ratio of 0.83 for any pterygium and a slightly smaller odds ratio of 0.92 for bilateral pterygium in multiple logistic regression models, but these data did not show a significant difference. Subjects with a history of outdoor jobs had slightly larger odds ratios of 1.22 for any pterygium and 1.95 for bilateral pterygium, but again the risk for pterygium did not reach a significant level. This may be because the towns of Minamiaizu-machi and Tadami-machi have heavy snowfall between November and March, and thus, outdoor work is difficult for about one-third of the year, which may reduce the risk of exposure to UV radiation.

The Kumejima Study indicated a significant difference in the prevalence of pterygium for subjects with a history of outdoor jobs. The yearly mean hours of sunlight in Kumejima is ≥1600, compared with about 1200 in the region in the current study. The residents of Minamiaizu-machi and Tadami-machi also tend to work in the early morning and evening, when UV radiation is weak. The fewer hours of sunlight and greater snowfall may lead outdoor workers in these areas to have lower cumulative exposure to UV radiation. This may also have caused the absence of a significant difference between the genders, although men may have a higher risk for the development of pterygium even after adjustment for exposure to UV radiation (McCarty et al. 2000; West & Munoz 2009). In this study, there may also have been an interaction between gender and outdoor job history. Thus, other factors may affect the relationship between gender and the risk for pterygium. Our data suggest that a more detailed evaluation of the association with UV radiation based on total working hours and protection against UV (McCarty et al. 2000) is needed in future studies.

Some studies have suggested that smokers are less likely to have pterygium (McCarty et al. 2000; Luthra et al. 2001), but smoking has been found to be a risk factor for pterygium among Chinese people (Wong et al. 2001). In the present study, smokers had a slightly higher odds ratio of 1.14 for any pterygium and slightly lower odds ratio of 0.75 for bilateral pterygium in multiple logistic regression models, but these effects were not significant. Induction of hormones or the immune system by smoking has been suggested (West & Munoz 2009), but further studies are needed to resolve this issue.

In conclusion, the prevalence of pterygium was 4.4% among subjects aged 40–74 years in Minamiaizu-machi and Tadami-machi, which are towns in north-eastern Japan. This is one of the lowest rates reported in a population-based study and lower than that in the Kumejima Study, which is the only previous Japanese population-based study. Age was associated with a risk of pterygium, but gender and outdoor job history were not associated with onset of pterygium in the current study.
